# Supplementary figures and images for: The optimisation of Salmonella surveillance programmes for pullet and layer farms using local farm density as a risk factor
Source: PLoS One. 2024 Apr 17;19(4):e0291896. doi: 10.1371/journal.pone.0291896 (PMC11023578; doi:10.1371/journal.pone.0291896)

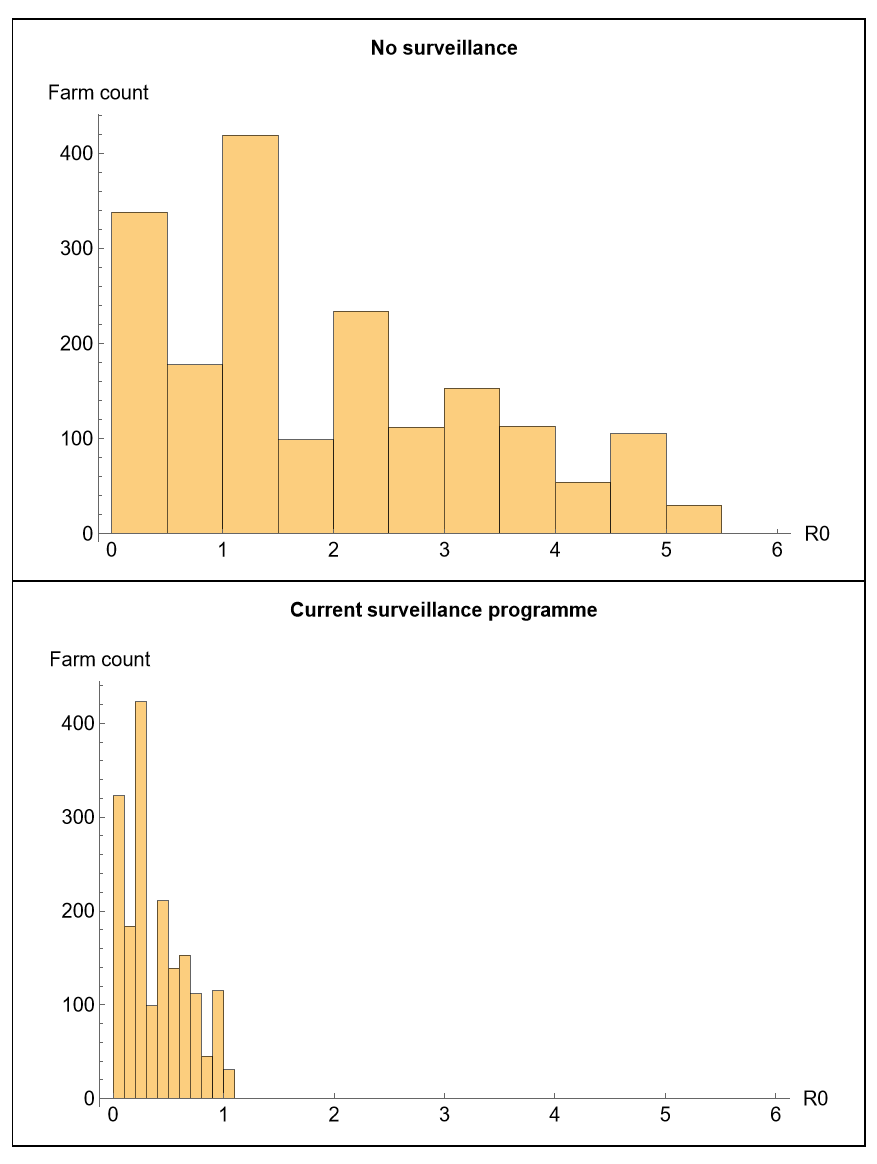

Supplement: S1 Fig — (TIF) [file pone.0291896.s001.tif]

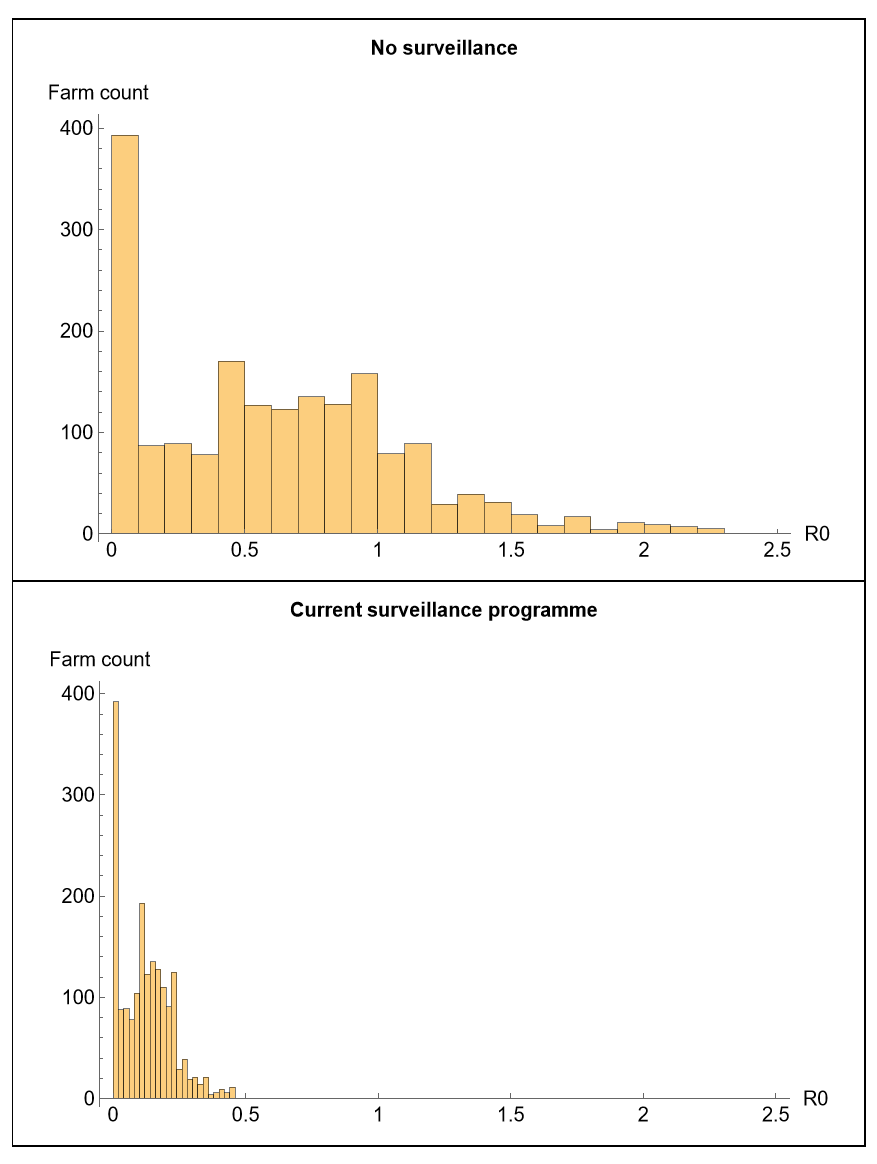

Supplement: S2 Fig — (TIF) [file pone.0291896.s002.tif]

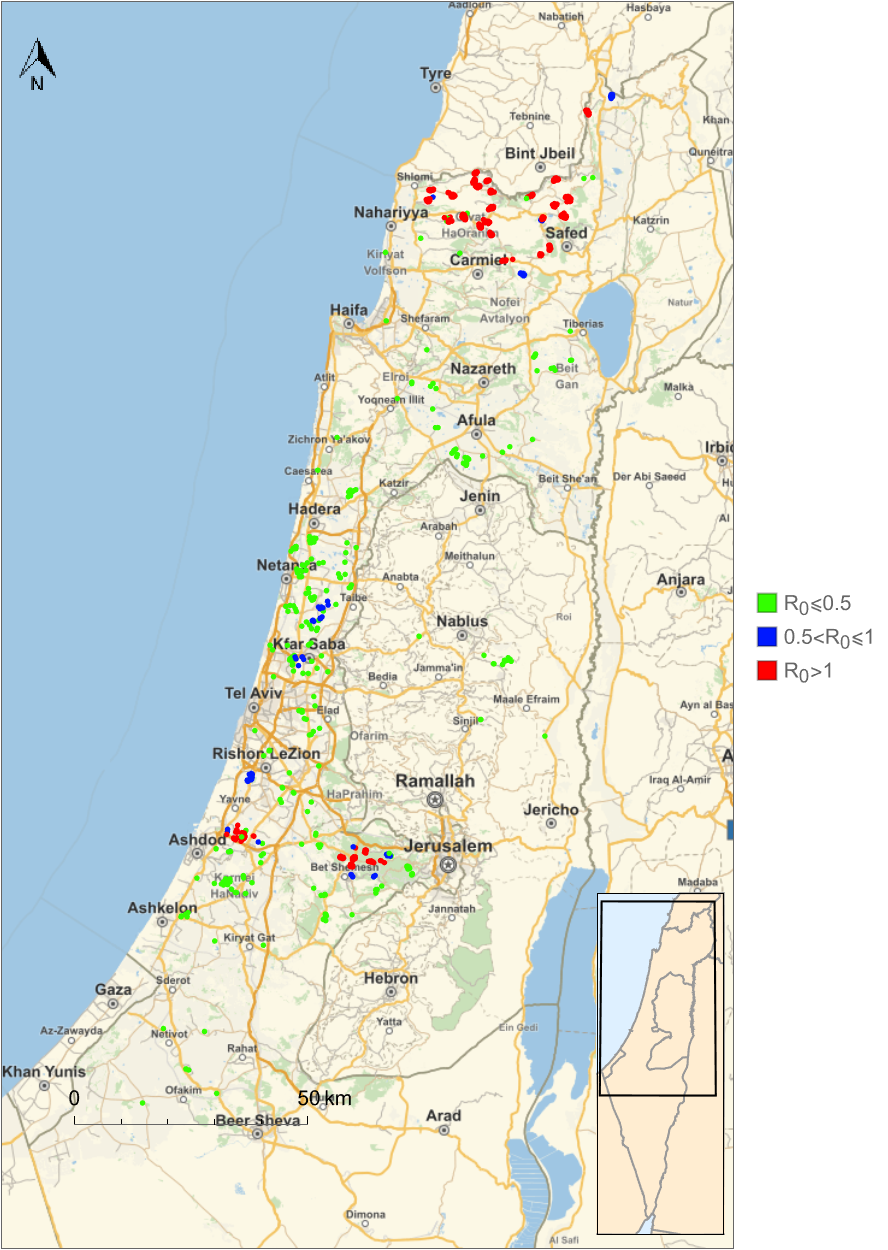

Supplement: S3 Fig — Base map from the Wolfram Knowledgebase and OpenStreetMap, which is made available under the Open Database License. (TIF) [file pone.0291896.s003.tif]

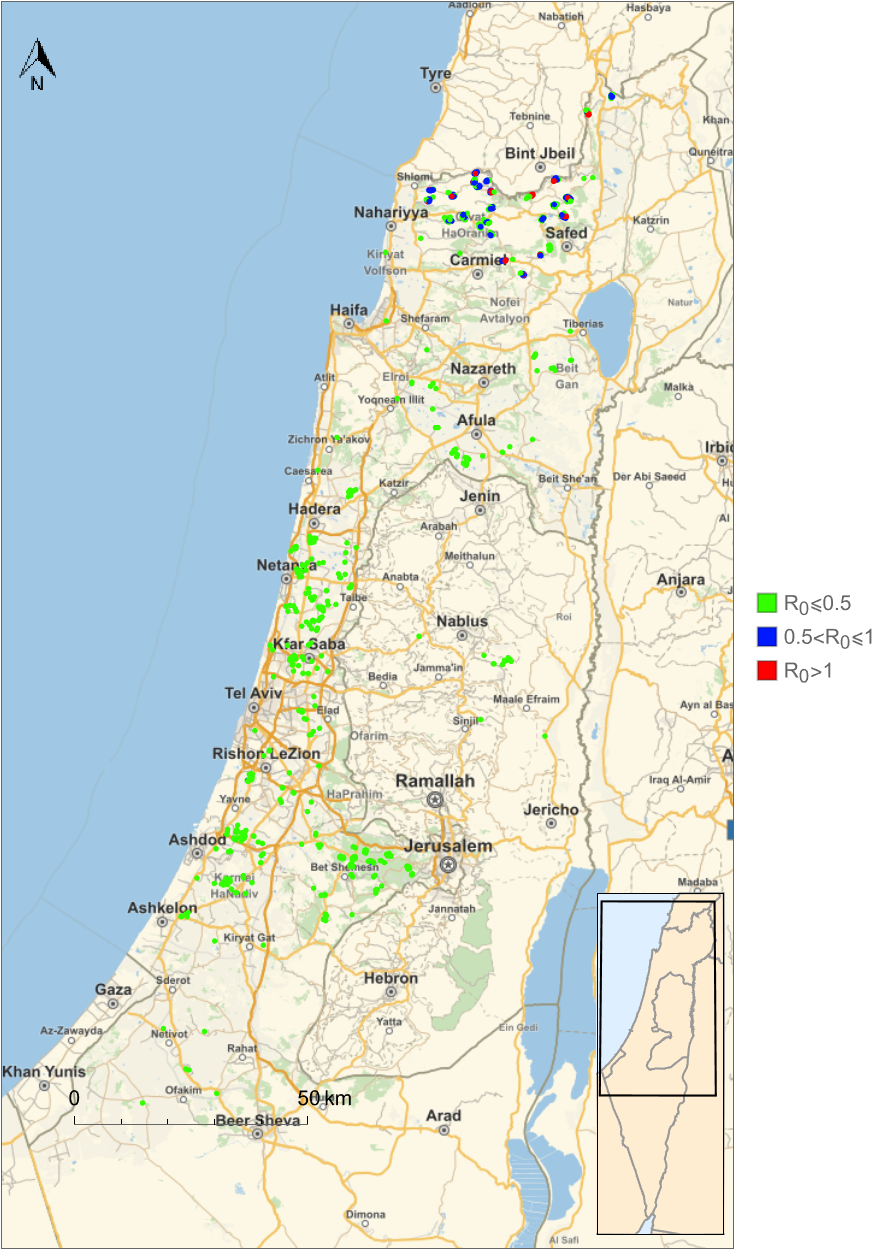

Supplement: S4 Fig — Base map from the Wolfram Knowledgebase and OpenStreetMap, which is made available under the Open Database License. (TIF) [file pone.0291896.s004.tif]
