# Supplementary material for: The optimisation of Salmonella surveillance programmes for pullet and layer farms using local farm density as a risk factor
Source: PLoS One. 2024 Apr 17;19(4):e0291896. doi: 10.1371/journal.pone.0291896 (PMC11023578; doi:10.1371/journal.pone.0291896)
Supplement: S1 Appendix — (DOCX) [file pone.0291896.s005.docx]

**S1 Appendix**

**Contents**

1. Reconstruction of the disease status of farms in time

2. Data cleaning

3. Missing information about positive flocks

4. Calculating the between-farm distance

**1. Reconstruction of the disease status of farms in time**

The original surveillance data were organised at the sample level with each row containing the information on one specific sample. In order to reconstruct the disease status of individual farms in time from the sample level data, we needed information on the variables listed in S1 Table (below) for each production cycle. Most of these variables were available in the dataset, except for the starting date of layer cycles and the date of infection (in case of positive samples). Data on the location of farms and the closing date of a production cycle were only available for a subset of all production cycles. If missing, we tried to estimate these variables using rules described below. If this was not possible, these production cycles were deleted from the data (see section 2 of this Appendix).

We reconstructed the disease status of farms in three steps. Firstly, we tried to reorganise the data from the sample level (one row for each sample) to the level of the production cycle (one row for each cycle). Secondly, we tried to reorganise the data from the level of the production cycle to the farm level (one row per farm). Thirdly, we used the farm level data to estimate the disease status of a farm in time. Below, we describe these different steps in more detail.

**Step 1: Reorganising the data from the sample level to the production cycle level**

At the end of this step, each row should contain information about the variables listed in S1 Table for a given production cycle. As mentioned above, some of these variables were not available and were estimated as described below (step 1A). When this was completed, we checked the chronological order of events within a production cycle and tried to resolve conflicting information (step 1B). Hereafter, samples that were taken during the sanitary period in between two production cycles are denoted as “Empty house samples” and samples that were taken during a production cycle when a flock was present are denoted as “Flock samples”. Also, we shortened “production cycle” to “cycle”.

Step 1A: Estimation of the starting and closing date of cycles if not available from the data

*Estimating the starting date of a cycle*

The starting date of a pullet cycle was present in the data. Hereafter, we will refer to this date as the “hatching date” of a flock. The starting date of layer cycles was estimated as 18 weeks past the hatching date of the flock.

*Estimating the closing date of a cycle*

Since cycles may be ongoing at the end of the data period, a closing date does not need to be present. In case there was no information on the closing date, but

1. the empty house was sampled after removal of the flock, the closing date was estimated as the day in the middle of the time period bounded by the last sampling date before flock removal and the date of the first empty house sample.
2. the empty house was NOT sampled after the flock was removed, but data on a subsequent cycle were present, we estimated the closing date in one of two ways. If the first sample belonging to the subsequent cycle was:
3. an empty house sample taken before introduction of the new flock, the closing date was estimated as described above under point 1.
4. a flock sample, the closing date was estimated as the date prior to the (estimated) day that the new flock was introduced.

Step 1B: Checking and resolving conflicts in the chronological order of events within a cycle

We checked for errors in the chronological order of sample types (Empty house/Flock sample) and mismatches between the starting, sampling and closing dates of a cycle. When trying to resolve mismatches between event dates, we assumed that starting dates of cycles were less reliable than closing dates and closing dates less reliable than sampling dates. Below we describe the different errors we checked for and how we tried to resolve them.

*Errors in the sequence of sample types*

We checked whether the sequence of empty house and flock samples made sense for a given cycle. In the sequences below, the term “Flock” may represent one or several consecutive flock samples and the term “Empty house” may represent one or several consecutive empty house samples. Using this notation, possible correct sequences are:

- Flock
- Flock – Empty house
- Empty house – Flock
- Empty house – Flock – Empty house

Incorrect sequences are:

- Empty house (ghost cycle)
- Flock – Empty house – Flock

We tried to merge ghost cycles (cycles only containing empty house samples) with the preceding cycle. Other cycles with an incorrect sequence of sampling types were deleted, except for cycles with positive samples for *Salmonella* serovars Enteritidis and/or Typhimurium. Hereafter, similar to the main text of the paper, we will refer to these serovars as SE and ST, respectively. We tried to find a specific solution for these cycles on a case-by-case basis.

*Mismatches between the starting and sampling dates of a cycle*

If the starting date of a cycle was estimated too early (when the house was still empty) or too late (after the flock was introduced), we adjusted the starting date using the rules described in S2 Table below.

*Mismatches between the sampling and closing dates of a cycle*

If the closing date of a cycle was estimated too early (when the flock was still present) or too late (after the empty house was sampled), we adjusted the closing date using the rules described in S3 Table below.

**Step 2: Reorganising the data from the production cycle level to the farm level**

Next, the data was formatted such that all information about one farm was on the same row in the data. We subsequently checked for overlap between consecutive production cycles. If this was the case, we tried to solve this by adjusting the starting date of the most recent cycle or by adjusting the closing date of the preceding cycle as described in S4 Table below. If this was not possible, we deleted the most recent cycle, except when it was positive for SE and/or ST. For the latter cycles, we found specific solution on a case-by-case basis.

**Step 3: Estimation of the time of infection**

The time of infection of a positive farm was estimated as described in S5 Table below.

**S1 Table.** Overview of information needed to reconstruct the disease status of farms in time and the availability of this information.

| **Variable name** | **Data available?** |
| --- | --- |
| Farm location | Yes, for a subset of production cycles |
| Farm Type (pullet/layer) | Yes |
| Starting date production cycle | For pullet farms only, not for layer farms. |
| Sampling dates | Yes |
| Sampling types (flock present or empty house) | Yes |
| Test results (diagnosis) | Yes |
| Infection date in case of positive test result | No |
| Closing production cycle (date flock was removed) | Yes, for a subset of production cycles |

**S2 Table.** Rules for adjusting the starting date of cycles in case of conflicts at the cycle level

| **Sequence of sampling types^a^:** | **Conflict** | **Starting date adjusted to:** |
| --- | --- | --- |
| Flock (- Empty House) | Starting date later than the first flock sample | One day before the first flock sample was taken. |
| Empty house – Flock  (- Empty House) | Starting date earlier than last empty house sample OR later than the first flock sample | The earliest of the two following dates:  - date last empty house sample plus the average # days between sampling and testing^b^  - one day before the first flock sample was taken |

^a^) Several consecutive samples of the same type may be taken. For example, if the sequence of sampling types is “Empty House – Flock”, several empty house samples may be followed by several flock samples.

^b^) The average time period from sampling to testing was set to 1 week. This time period was used because a new flock cannot be introduced into a house until a negative test result.

**S3 Table.** Rules for adjusting the closing date of cycles in case of conflicts at the cycle level

| **Sequence of sampling types^a^:** | **Conflict** | **Closing date adjusted to:** |
| --- | --- | --- |
| Flock OR  Empty house - Flock | Closing date earlier than the last flock sample | One week after the last flock sample was taken |
| Flock - Empty house OR  Empty house – Flock – Empty house | Closing date earlier than the last flock sample OR  later than the first empty house sample after the flock was removed | The day in the middle of the period between the last flock sample and the first empty house sample |

^a^) Several consecutive samples of the same type may be taken. For example, if the sequence of sampling types is “Empty House – Flock”, several empty house samples may be followed by several flock samples.

^b^) The average time period from sampling to testing was set to 7 days. This time period was used because a new flock cannot be introduced into a house until a negative test result.

**S4 Table.** Rules for adjusting the starting or closing date of cycles in case of conflicts at the farm level

| **Preceding cycle ending with sampling type:** | **Current cycle starting with sampling type** | **Conflict** | **Conflict resolved by:** |
| --- | --- | --- | --- |
| Flock or Empty House | Flock | Starting date cycle earlier than closing date or last empty house sample from the preceding cycle | Adjusting the starting date of the current cycle to 1 day after the closing date or last empty house sample of the preceding cycle |
| Flock | Empty House | Closing date preceding cycle later than the date of the first empty house sample from the current cycle | Adjusting the closing date of the preceding cycle to 1 day before the first empty house sample of the current cycle was taken |

**S5 Table.** The rules for estimating the date of infection of farms by *Salmonella* Enteritidis and Typhimurium from the available data.

| **Scenario** | **Method for estimating the date of infection** |
| --- | --- |
| - The first positive sample was not the first sample taken during a given cycle | The date in the middle of the interval between the last negative and first positive sample |
| - The first positive sample was also the first sample taken during a cycle  AND  - the starting date of the cycle was located within the period for which surveillance data was available | The date in the middle of the interval between the start of the cycle and the first positive sample |
| - The first positive sample was also the first sample taken during a cycle  AND  - the start of the cycle preceded the period for which surveillance data was available | The latest of the two following dates:  - the middle of the interval between the start of the cycle and the first positive sample  - the middle of the interval between the start of the period for which surveillance data is available and the first positive sample |

**2. Data cleaning**

The original surveillance data were organised at the level of individual samples. In order to reconstruct the disease status of farms in time, we reorganised these data at the level of the individual production cycles and subsequently at the farm level. At each organisation level, we performed a number of data cleaning steps, which are described below. The number of farms and production cycles that were excluded from the analysis and the reason for excluding them are given in S6 to S8 Tables.

**Data organised at the level of individual samples**

**-** Deletion of duplicate rows.

- Deletion of rows containing fields with impossible or incorrectly formatted content.

**Data organised at the level of individual production cycles**

- Deletion of cycles for which required information (see S1 Table) was missing that could not be estimated using the methods described above (section 1 of this Appendix).

- Deletion of cycles with an incorrect chronological sequence of sampling types, with the exception of cycles with positive samples for SE and/or ST.

**Data organised at the level of individual farms**

- In case two cycles were overlapping and this could not be resolved by adjusting starting or closing dates as described above (section 1 of this Appendix), the most recent cycle was deleted, except when it was positive for SE and/or ST.

**S6 Table.** The number of excluded production cycles and the reason for their exclusion.

| **Reason for excluding a production cycle** | **Number of excluded production cycles** |
| --- | --- |
| Farm location missing | 65 |
| Hatching date of flock missing | 6 |
| Error in the sequence of sampling types (Empty house sample or flock sample) | 9 |
| Overlap between cycles | 6 |

**S7 Table.** The number of excluded farms and the reason for their exclusion.

| **Reason for excluding a farm** | **Number of excluded farms** |
| --- | --- |
| Farm location missing | 54 |
| Hatching date of flock missing | 4 |
| Error in the sequence of sampling types (Empty house sample or flock sample) | 2 |
| Overlap between cycles | 3 |

**S8 Table.** The number of farms included in the analysis for which one or more production cycles were excluded and the reason for exclusion of these cycles.

| **Number of farms included in the analysis but with one or more cycles excluded** | **Reason for excluding a production cycle** |
| --- | --- |
| 2 | Hatching date of flock missing |
| 7 | Error in the sequence of sampling types (Empty house sample or flock sample) |

**3. Missing information about positive flocks**

After the analyses were completed, a number of additional flocks were found to be positive for SE/ST during the study period. Some of these flocks were completely missing from the analysed data, while others were present but assumed to be negative for SE/ST. The S9 Table below shows the different reasons why information about flocks was missing from the analysed data and the number of affected flocks.

**S9 Table.** The reason why flocks, that were infected by SE/ST, were missing from the analysed data or were assumed to be negative for SE/ST.

| **Reason for incomplete flock data** | **Number of affected flocks positive for SE** | **Number of affected flocks positive for ST** |
| --- | --- | --- |
| Both farm and flock were missing from data | 0 | 1 |
| Farm was present, but flock was missing from data | 2 | 0 |
| Flock was present in data, but *Salmonella* samples were identified to the group level at the time of the analyses and only later identified to the serovar level. | 1 | 1 |
| Farm coordinates for positive flock were initially missing | 0 | 1 |

**4. Calculating the between-farm distance**

Between-farm distances were derived from available cartesian coordinates. We noticed that the distance between two farms was very small for certain farm combinations with the smallest distance amounting to just 6 m. Since this did not seem realistic, we set the minimum between-farm distance to 50 meters. This minimum distance replaced the calculated between-farm distance for 0.06% of all possible unique combinations of two farms.
